# flowVS: channel-specific variance stabilization in flow cytometry

**DOI:** 10.1186/s12859-016-1083-9

**Published:** 2016-07-28

**Authors:** Ariful Azad, Bartek Rajwa, Alex Pothen

**Affiliations:** 1 Computational Research Division, Lawrence Berkeley National Laboratory, 1 Cyclotron Rd, Berkeley, 94720 CA USA; 2 Bindley Bioscience Center, Purdue University, West Lafayette, 47907 IN USA; 3 Department of Computer Science, Purdue University, West Lafayette, 47907 IN USA

**Keywords:** Variance stabilization, Flow cytometry, Bartlett’s test, Microarrays

## Abstract

**Background:**

Comparing phenotypes of heterogeneous cell populations from multiple biological conditions is at the heart of scientific discovery based on flow cytometry (FC). When the biological signal is measured by the average expression of a biomarker, standard statistical methods require that variance be approximately stabilized in populations to be compared. Since the mean and variance of a cell population are often correlated in fluorescence-based FC measurements, a preprocessing step is needed to stabilize the within-population variances.

**Results:**

We present a variance-stabilization algorithm, called flowVS, that removes the mean-variance correlations from cell populations identified in each fluorescence channel. flowVS transforms each channel from all samples of a data set by the inverse hyperbolic sine (asinh) transformation. For each channel, the parameters of the transformation are optimally selected by Bartlett’s likelihood-ratio test so that the populations attain homogeneous variances. The optimum parameters are then used to transform the corresponding channels in every sample. flowVS is therefore an explicit variance-stabilization method that stabilizes within-population variances in each channel by evaluating the homoskedasticity of clusters with a likelihood-ratio test.

With two publicly available datasets, we show that flowVS removes the mean-variance dependence from raw FC data and makes the within-population variance relatively homogeneous. We demonstrate that alternative transformation techniques such as flowTrans, flowScape, logicle, and FCSTrans might not stabilize variance. Besides flow cytometry, flowVS can also be applied to stabilize variance in microarray data. With a publicly available data set we demonstrate that flowVS performs as well as the VSN software, a state-of-the-art approach developed for microarrays.

**Conclusions:**

The homogeneity of variance in cell populations across FC samples is desirable when extracting features uniformly and comparing cell populations with different levels of marker expressions. The newly developed flowVS algorithm solves the variance-stabilization problem in FC and microarrays by optimally transforming data with the help of Bartlett’s likelihood-ratio test. On two publicly available FC datasets, flowVS stabilizes within-population variances more evenly than the available transformation and normalization techniques. flowVS-based variance stabilization can help in performing comparison and alignment of phenotypically identical cell populations across different samples. flowVS and the datasets used in this paper are publicly available in Bioconductor.

## Background

We describe an algorithm that transforms a collection of flow cytometry (FC) samples in order to stabilize the variance within cell populations in each fluorescence channel for the entire collection of samples. This transformation enables cell populations (clusters of cells with similar phenotypes) with homogeneous variances to be easily compared with each other by standard statistical methods. Between-population comparisons are important in detecting changes in populations across biological conditions, which might help us to diagnose diseases, develop new drugs, and understand the immune system in general [1–5]. Hence, our variance-stabilization algorithm could play a supporting role in automating biological discovery based on flow cytometry and similar imaging technologies.

FC technology measures morphology (from light scattering) and the expression of multiple biomarkers (from fluorescence emission of fluorophores attached to antibodies) at the single-cell level. An FC sample consists of hundreds of thousands or more of such single-cell measurements, and a study could consist of thousands of samples from different individuals at different time points under different experimental conditions [6, 7].

**Fig. 1:**
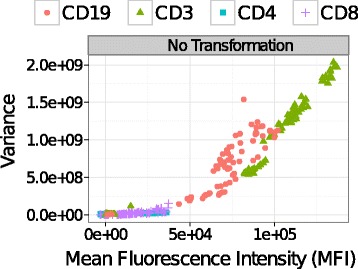
Mean fluorescence intensities (MFIs) of one-dimensional cell populations (also called density peaks) are plotted against the variances of the populations. Blood samples were collected from five healthy individuals on different days and stained with labeled antibodies against five biomarkers (see Section 3). Samples are compensated and gated for the lymphocytes, but no transformation is used. Populations identified in each fluorescence channel are shown with the same symbol and color. We observe that without proper transformation, variance increases monotonically with MFI

Variance inhomogeneity is an inherent problem in fluorescence-based FC measurements and can be an obstacle both for manual data analysis performed by qualified cytometry operators and for automated multi-sample comparisons, which typically rely on an intermediate step of cell clustering using a plethora of approaches from modified k-means to non-parametric Bayesian methodologies [8, 9]. The origin of the problem is the physics of fluorescence signal formation and the detection processes that monotonically increase the variance of the fluorescence signal with the average signal intensity [10, 11]. For example, Fig. 1 demonstrates how the variances of cell populations increase with their mean fluorescence intensities (MFIs) in a set of FC samples collected from several healthy individuals. Owing to such signal-variance dependence, a cell population with higher levels of marker expressions (i.e., higher fluorescence emission) has higher variance than another population with relatively low levels of marker expressions (i.e., low fluorescence emission). This inhomogeneity of within-population variance creates problems in extracting features uniformly and comparing cell populations with different levels of marker expressions.

In order to demonstrate the flowVS results we evaluate the pre- and post-processing cluster homogeneity, and quantify the improvement offered by our approach. We report the results using a simple measure of effect size, rather than through a hypothesis-testing framework. As an example, consider the population registration problem in which corresponding cell clusters from multiple FC samples are identified based on the average levels of markers expressed by the clusters [3, 12–14]. The clusters of cells representing the same immunophenotype identified in multiple samples are represented by a hypothetical metacluster (a biological generalization of the particular immunophenotype, observed across multiple samples). The existence of metaclusters is typically assessed by hemopathologists or other skilled FC operators on the basis of their experience and knowledge of previous examples of normal and aberrant immunophenotypes. The biological hypothesis behind assigning a cluster to a metacluster can be formulated as “all clusters in a metacluster represent the same cell type (immunophenotype).” However, translating this hypothesis to a null stating “all clusters in a metacluster have equal mean” and using a traditional hypothesis-testing framework accompanied by *p*-values may not be appropriate. First, we know that such a null hypothesis is unrealistic: biological variability, technical variability of blood or bone-marrow sample measurements, and random effects associated with the biochemistry of antibody binding will certainly produce clusters of differing means. Second, a hypothesis-testing framework addresses only the question of whether the clusters have the same location, but it is not designed to measure the magnitude of the difference or lack of homogeneity within a postulated metacluster. Finally, the *p*-values are affected by both cluster size and metacluster homogeneity. Thus, the *p*-values obtained would not be comparable for various metaclusters or different clusters within metaclusters.

Variance stabilization (VS) is a process for dissociating data variability from mean signals [15–18]. Other fluorescence-based technologies such as the microarrays stabilize variance by data transformation [18–21]. However, unlike microarray data, explicit VS is not usually performed in FC data analysis. Traditionally, FC data are transformed with nonlinear functions to project cell populations with normally distributed clusters – a choice that usually simplifies subsequent visual analysis [22–27]. Recently, Finak et al. [27] used the maximum-likelihood approach to explicitly satisfy normality of the cell populations. Ray et al. [28] transformed each channel with the asinh function whose parameters are optimally selected by the Jarque-Bera test of normality (a goodness-of-fit test of whether sample data have the skewness and kurtosis matching a normal distribution). While these transformations approximately normalize FC data, they might not stabilize variance, as may be seen in Figs. 6 and 7.

The VS problem in FC, however, cannot be solved directly by applying mature VS techniques from the microarray literature. In microarrays, each gene is measured multiple times (possibly under multiple conditions) and the between-sample variance for each gene is stabilized with respect to the average expression of the gene across samples. By contrast, variance is defined by within-population cell-to-cell variation in FC, and this within-population variance is stabilized with respect to the average expression of markers within each population. These contrasting objectives prevent us from applying VS methods from microarray literature directly to flow cytometry.

We address the need for explicit VS in FC with a maximum likelihood (ML)-based method, called flowVS, which is built on top of a commonly used inverse hyperbolic sine (asinh) transformation. The choice of asinh function is motivated by its success as a variance stabilizer for microarray data [18, 21]. flowVS stabilizes the within-population variances separately for each fluorescence channel *z* across a collection of *N* samples. After transforming *z* by asinh(*z*/*c*), where *c* is a normalization *cofactor*, flowVS identifies one-dimensional clusters (density peaks) in the transformed channel. Assume that a total of *m* 1-D clusters are identified from *N* samples with the *i*-th cluster having variance ${\sigma ^{2}_{i}}$. Then the asinh(*z*/*c*) transformation works as a variance stabilizer if the variances of the 1-D clusters are approximately equal, i.e., ${\sigma ^{2}_{1}} \sim {\sigma ^{2}_{2}}\sim \ldots \sim {\sigma ^{2}_{m}}$. To evaluate the homogeneity of variance (also known as homoskedasticity), we use Bartlett’s likelihood-ratio test [29]. From a wide range of cofactors, our algorithm selects one that minimizes Bartlett’s test statistic, resulting in a transformation with the best possible VS. Note that, in contrast to other transformation approaches, our algorithm applies the same transformation to corresponding channels in every sample. flowVS is therefore an explicit VS method that stabilizes within-population variances in each channel by evaluating the homoskedasticity of clusters with a likelihood-ratio test.

Using a healthy-subject data set from Purdue and publicly available immune tolerance network (ITN) data, we demonstrate that flowVS removes the mean-variance dependence from raw FC data and makes the within-population variance relatively homogeneous. We demonstrate that alternative transformation techniques might not stabilize variance. Variance homogeneity is especially useful to build metaclusters from a collection of phenotypically similar cell populations across samples [3, 27, 30, 31]. Previous studies (Hahne et al. [32], for example) shifted the distribution of each fluorescence channel to ensure homogeneity in metaclusters, but such shifting might hide useful biological signals present in the MFIs of cell populations. By contrast, we can build homogeneous metaclusters from variance-stabilized populations without removing the differences in their MFIs. Hence, flowVS could provide additional flexibility in processing and analyzing a large collection of FC samples.

## Related work

VS has been a widely studied topic in applied statistics for its central role in making heteroskedastic data easily tractable by standard methods. Heteroskedasticity appears in various data sets mostly because the data follow a distribution with correlated mean and variance, e.g., Poisson or Gamma; there are many more examples, but these two are relevant for fluorescence. For well-known distribution families, VS is usually performed by transforming data with an analytically chosen function *f*. For example, $f(z)=\sqrt {z+3/8}$ works as a good (asymptotic) stabilizer for a random variable *z* following the Poisson distribution [33]. Variance stabilizers for several well-known distribution families are described in [33, 34]. For unknown distributions, heuristic and data-driven stabilizers are often used [15–17].

However, traditional transformations are often inadequate for low-count (photon-limited) signals [18, 35] because of unknown error patterns in fluorescence data. Past work developed ad hoc VS schemes for different types of fluorescence data. For example, in microarrays, the VS problem has been addressed by various non-linear transformations [18–21]. Most notably, the widely used approach by Huber et al. [18] uses an asinh transformation whose parameters are selected by a maximum-likelihood estimation.

For FC data, researchers have used various non-linear transformations, such as the logarithm, hyperlog, generalized Box-Cox, and biexponential (e.g., logicle and generalized arcsinh) functions [22–27]. In past work, parameters of these transformations were adjusted in a data-driven manner to maximize the likelihood (flowTrans by Finak et al. [27]), to satisfy the normality (flowScape by Ray et al. [28]), and to comply with simulations (FCSTrans by Qian et al. [36]). flowTrans estimates transformation parameters for each sample by maximizing the likelihood of data’s being generated by a multivariate-normal distribution on the transformed scale. flowScape optimizes the normalization factor of asinh transformation by the Jarque-Bera test of normality. FCSTrans selects the parameters of the linear, logarithm, and logicle transformations with an extensive set of simulations. However, normalizing data may not necessarily stabilize its variance, e.g., for a Poisson variable *z*, $\sqrt {z+3/8}$ is an approximate variance-stabilizer, whereas *z*^2/3^ is a normalizer [16]. Therefore, we consider an approach built upon the well-known asinh transformation and estimate transformation parameters for explicitly stabilizing within-population variations.

## Methods

### Motivation

**Fig. 2:**
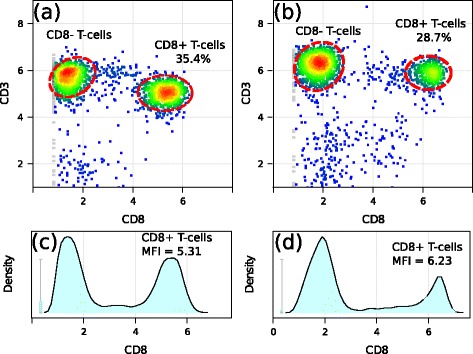
Subfigs. (**a**) and (**b**) show the 2D-projections of T-cell subpopulations from two samples in the ITN data set. Distributions of CD8 marker are shown below the corresponding samples in Subfigs. (**c**) and (**d**)

Consider two representative samples from the ITN data set taken from the flowStats package in Bioconductor. We gate the samples for lymphocytes, transform using an asinh transformation (with cofactor set to 1), and plot T-cell subpopulations and distribution of CD8 marker in Fig. 2. Across these two samples, T-cell subpopulations have different proportions and MFIs. For example, in Subfig. 2a and 2c, CD8^+^ T-cell subpopulation is 35.4 % of total T-cells with MFI 5.31. By contrast, CD8^+^ T-cell subpopulation is 28.7 % of total T cells with MFI 6.23 in Subfig. 2b and 2d. Should we consider the differences between CD8^+^ populations in these two samples to be biologically significant? The answer to this question depends on our assumption about the data. If we assume that a cell type either expresses or does not express a biomarker and that the biological information lies only in the proportion of positively and negatively expressed cells, then MFI does not bear meaningful information other than defining positive and negative cells. In this case, we could consider the differences in MFIs across cell populations of the same type as technical variations and eliminate them by aligning cell populations described by Hahne et al. [32] and Finak et al. [37]. However, past work has shown that both cell proportion and MFI can possess biological information [2, 3, 38]. Hence, aligning cell populations to a common MFI might remove meaningful biological signal from data. In the latter case, we want to compare MFIs of cell populations to evaluate whether they are statistically different. A common statistical approach to compare average expressions of cell population is to use a statistical test in an ANOVA model that explicitly requires that variance be approximately stabilized in populations. Hence, VS is necessary to detect statistically meaningful changes across populations from different samples.

### The goal of VS in flow cytometry

The aim of VS in FC is to make within-population variances of different cell populations approximately equal and thereby independent of the average marker expressed by populations. Recall that the expression of a marker is measured by the intensity of light at a particular channel of fluorescence. VS therefore stabilizes the within-population fluorescence variance and makes it independent of the MFIs of the cell populations. In this paper, we refer to fluorescence channels more frequently because the nature of fluorescence emissions – not the protein expressions – dominates the mean-variance relationship in FC data. We do not stabilize variance on the scatter channels because, as pointed out by Finak et al. [27], there are few benefits to transforming forward- and side-scatter channels.

### Channel-specific variance stabilization

We assume that correlations among fluorescence channels due to the overlap of spectra are removed by spectral unmixing before we transform data. Even though the expression of biomarkers can still be correlated [24], we do not incorporate such correlations in VS because the nature of such correlation is difficult to model. Therefore, we assume that compensated fluorescence channels are independent and stabilize variance on each channel separately.

Selecting an optimal transformation for FC data is a nontrivial problem because the accurate error model of FC data is often unknown. In previous work, researchers have successfully used a number of functions to transform FC data, such as logarithm, asinh, Box-Cox, logicle, etc. [24, 27, 28]. In our flowVS algorithm, we decided to use the asinh function to transform FC data. This choice of asinh function is motivated by its success in FC data visualization and normalization [27, 28] and in stabilizing variance in fluorescence readouts from microarray data [18, 21]. Stabilizing variance with other transformations can be performed using the same flowVS framework but is not discussed here.

To transform a fluorescence channel *z*, we use the asinh transformation with a single parameter *c*:

1 $$ \operatorname{asinh} (z/c) = \ln(z/c + \sqrt{(z/c)^{2} + 1}).   $$

In this transformation, *c* is called the normalization cofactor, whose value is optimally selected to stabilize within-population variance in channel *z*. Note that in a more general form asinh transformation is expressed with three parameters, *a*∗asinh(*b*+*z*/*c*), where in addition to the cofactor *c*, *a* denotes a scaling after transformation, and *b* denotes a translation before transformation. We set *a*=1 because scaling after transformation does not affect downstream analysis and set *b*=0 to avoid shifting cell populations. Hence, we are left with a single parameter *c* whose value is estimated in order to stabilize the variance.

### The flowVS algorithm

Assume that we have a collection of *N* FC samples. Then the objective of the flowVS algorithm is to transform each sample such that the within-population variance is stabilized in each fluorescence channel across *N* samples. Here, we describe the algorithm for a single channel *z*; the process can be applied independently to other channels. First, we discuss the process of evaluating homoskedasticity of a transformed channel for a selected cofactor *c* by computing Bartlett’s likelihood-ratio test. Then, we elaborate the process of selecting an optimum cofactor that would stabilize variance when used with asinh transformation.

#### Steps to compute Bartlett’s statistic on channel *z* for a selected cofactor *c*

- **Step1:***Transforming channel z in each sample.* Let *z*_*j*_ be a vector denoting channel *z* in the *j*-th sample, where 1≤*j*≤*N*. We transform *z*_*j*_ by the asinh function: $z^{\prime }_{j} = \operatorname {asinh} (z_{j}/c)$, where $z^{\prime }_{j}$ is the transformed channel.
- **Step2:***Detecting 1-D density peaks (1-D clusters)*. We estimate the density of $z^{\prime }_{j}$ by a kernel density estimation method (the density function of stats package in R). The peaks in the density of $z^{\prime }_{j}$ are identified as regions of high local density and significant curvature (also called landmarks in [32]). We identify high-density regions in $z^{\prime }_{j}$ by the curv1Filter function of the flowCore package [39] in Bioconductor. The boundaries of density peaks are identified by detecting minima between two adjacent density peaks. Here, a density peak represents a 1-D cluster of cells. Let *P*_*j*_ be the collection of all density peaks identified in $z^{\prime }_{j}$.
- **Step3:***Collecting density peaks from all samples.* Let *P* be the set of density peaks collected from all samples, i.e., *P*=∪_1≤*j*≤*N*_*P*_*j*_. Let *P* contain a total of *m* density peaks where the *i*-th peak contains *n*_*i*_ cells with mean *μ*_*i*_ and variance ${\sigma ^{2}_{i}}$.
- **Step4:***Computing Bartlett’s test statistic.* Let $n=\sum _{1\leq i \leq m} n_{i}$ be the total number of cells in *P* and ${\sigma ^{2}_{p}}$ be the pooled variance of *m* density peaks. Then we compute Bartlett’s statistic as follows:
  2 $$ B(c) = \frac{(n-m)\: \ln\left({\sigma_{p}^{2}}\right) - \sum_{i=1}^{m} (n_{i}-1)\: \ln\left({\sigma_{i}^{2}}\right)}{1 + \frac{1}{3(m-1)} \left(\sum_{i=1}^{m} \frac{1}{n_{i}-1} - \frac{1}{n-m}\right)}.   $$
  This statistic *B*(*c*) is specific to the cofactor *c* used to transform the data and measures the degree of homogeneity across all 1-D clusters in the transformed channel *z*^′^.

#### Finding a cofactor for optimum VS

The optimum variance-stabilizing cofactor *c*^∗^ is a cofactor giving the minimum value of Bartlett’s statistic:

3 $$ c^{*} = \arg\!\min B(c).   $$

Minimizing Eq. 2 is a nontrivial optimization problem because Bartlett’s test statistic *B*(*c*) depends indirectly on the cofactor *c* and is not differentiable with respect to *c*. This prevents us from applying optimization methods from the gradient-descent family. Therefore, we employ a piecewise minimization without derivatives [40]. Let *c*_*low*_ and *c*_*high*_ be the lowest and highest possible values of the cofactor on a logarithmic scale. By default, we set *c*_*low*_=−2 and *c*_*high*_=10, i.e., the lowest and highest values of cofactor is exp(−2)∼0.135 and exp(10)∼22026, respectively. Users can also supply these extreme values. We assume that the optimum cofactor lies in the range [ exp(*c*_*low*_), exp(*c*_*high*_)]. Then the optimization procedure works as follows:

- (a) We divide the interval [ *c*_*low*_, *c*_*high*_] into *k*=(*c*_*high*_−*c*_*low*_) equal regions where the *i*-th region is defined by the interval [*c*_*i*_,*c*_*i*+1_] and *c*_*i*+1_−*c*_*i*_=1.
- (b) For the *i*-th interval, we look for a cofactor in the range [ exp(*c*_*i*_), exp(*c*_*i*+1_)] with minimum Bartlett’s statistic. For each cofactor, we compute the Bartlett’s statistic with the steps described in Sec. 3. For faster convergence, we call the optimize function from the stats package in R, which uses a combination of golden section search and successive parabolic interpolation [41]. Interested readers might see the R documentation for a detailed description of the function. Let $c^{*}_{i}$ be the optimum cofactor in the *i*-th interval with the associated Bartlett’s statistic $B(c^{*}_{i})$.
- (c) We identify the overall optimum cofactor *c*^∗^ as follows:
  4 $$ c^{*} = \arg\!\min_{i=1}^{k} B(c^{*}_{i}).   $$

Equation 4 provides an approximate solution to Eq. 3. Since we divided the search space into smaller intervals, the probability of having multiple local optima in an interval is small. Hence, the procedure described above is expected to return a variance stabilizing cofactor. After we obtain the optimum cofactor *c*^∗^, channel *z* in each sample is transformed by asinh(*z*_*j*_/*c*^∗^) and used in subsequent analysis.

## Results

### Data sets

We demonstrate the use of flowVS and other related methods by using a healthy-subject data set from Purdue University (HD) and publicly available immune tolerance network (ITN) data. The original HD data set consists of 65 samples from five healthy individuals who donated blood on different days [42]. Here, for simplicity, we used a smaller subset of the HD data set consisting of 12 samples from three healthy individuals, “A”, “C”, and “D”. From each individual, we keep samples from two (randomly selected) days and two technical replicates from each day. Each HD sample was stained using labeled antibodies against CD45, CD3, CD4, CD8, and CD19 protein markers. In this paper, an HD sample “C_4_2” means that it is collected on day 4 from individual “C” and it is the second replicate on that day. The healthy data set is part of our Bioconductor package flowVS. The ITN data set is collected from 15 patients. It includes 3 patient groups with 5 samples each. Each sample was stained using labeled antibodies against CD3, CD4, CD8, CD69 and HLADr. The ITN data set is available in the flowStats package in Bioconductor. We selected these data sets because they are available in standard R packages. Hence, the results presented here can be easily reproduced.

**Fig. 3:**
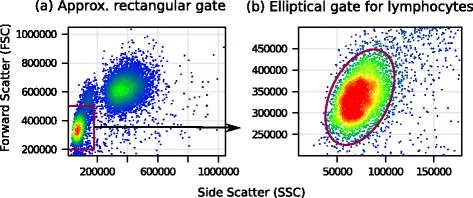
Identifying lymphocytes by a two-step gating from a representative sample in the HD data set. **a** We select an approximate rectangular region in the lower left corner of side-scatter vs. forward-scatter plot. **b** A dense elliptical region within the rectangular gate defines lymphocytes

We identify lymphocytes in each sample of the HD and ITN datasets by using a two-step gating shown in Fig. 3. In this paper, we perform data transformation only on lymphocytes.

### Stabilizing variance in the HD dataset

**Fig. 4:**
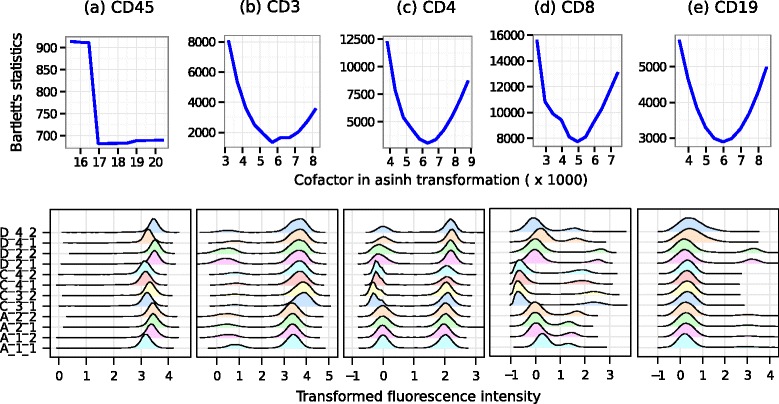
Transforming five fluorescence channels in HD data. Subfigures in the top row show Bartlett’s statistic computed from density peaks after data are transformed by different cofactors. An optimum cofactor is obtained where Bartlett’s statistic reaches the minimum. The bottom row shows the density plots after the data are transformed by an asinh transformation with the optimum cofactors

At first, flowVs identifies an optimum cofactor for asinh transformation for each fluorescence channel of the HD dataset. This process is performed by identifying density peaks in each channel and minimizing Bartlett’s statistic, as described in Section 3. The top row in Fig. 4 shows Bartlett’s statistic computed from density peaks of all samples after the data are transformed by asinh transformation with different cofactors. The range of values of the cofactor is selected by the automated algorithm described in Section 3. An optimum variance-stabilizing cofactor is obtained where Bartlett’s statistic is a minimum. From Fig. 4, the variance-stabilizing cofactors for different markers are: (a) 17,956 for CD45, (b) 5,685 for CD3, (c) 6,317 for CD4, (d) 4,937 for CD8, and (e) 5,976 for CD19. For every channel except CD45, we obtain a clear global minimum, denoting the existence of a unique variance-stabilizing cofactor with respect to Bartlett’s test. For CD45, we observe a sharp decrease in Bartlett’s statistic at cofactor 17,000. Since CD45 is a common leukocyte marker, it is always expressed on lymphocytes – the subset of cells that we preselected for this study. Hence, most cells are CD45+ in our preprocessed samples, which might produce a non-convex relationship between Bartlett’s statistic and cofactors. For the same reason, the value of Bartlett’s statistic at the optimum cofactor for CD45 is the smallest (less than 700) compared to the minimum value of Bartlett’s statistic achieved in other channels. Note that the minimum Bartlett’s statistic denotes the degree to which we are able to stabilize the within-population variance of a channel considering the between-sample variations.

We transform each sample of the HD data set by the asinh function with the variance-stabilizing cofactors and plot the density of the transformed channels in the bottom row of Fig. 4. In each channel, we observe that density peaks (a.k.a. one-dimensional clusters) have approximately equal width across all samples, which visually confirms the homogeneity of within-population variances in one-dimensional clusters. When both positive and negative peaks (i.e., clusters with high or low marker expression) are present in a channel, e.g., CD3, CD4, and CD8, their variances are also approximately stabilized. Note that the density peaks may not be well aligned owing to the between-subject variations. Aligning density peaks across samples is not an objective of flowVS, because such shifting of density might potentially eclipse biological signals present in the mean expressions of a cell populations. When necessary, data normalization can be performed after variance stabilization, as was done by Hahne et al. [32] and Finak et al. [37].

### Stabilizing variance in the ITN dataset

**Fig. 5:**
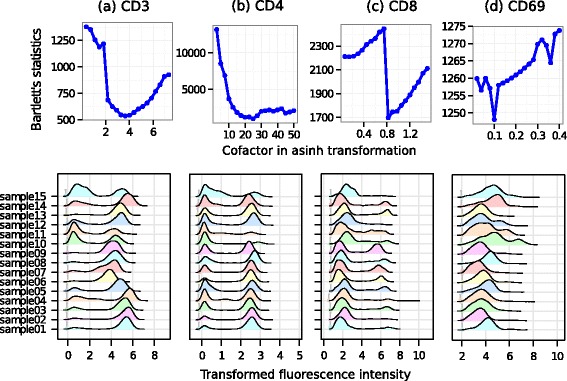
Transforming four fluorescence channels in ITN data. Subfigures in the top row show Bartlett’s statistic computed from density peaks after data are transformed by different cofactors. An optimum cofactor is obtained where Bartlett’s statistic reaches the minimum. The bottom row shows the density plots after the data are transformed by the optimum cofactor

We stabilize variance in each channel of the ITN dataset and show the results in Fig. 5. Similar to the HD data set, the top row shows Bartlett’s statistic computed from density peaks of all samples of the ITN data set after each channel is transformed by asinh transformation with different cofactor for each one. From Fig. 5, the variance stabilizing cofactors for different markers are: (a) 3.66 for CD3, (b) 25.1 for CD4, (c).75 for CD8, and (d) 0.2 for CD69. The curves showing the relationship between Bartlett’s statistic and cofactors might have multiple local minima. Nevertheless, a clear global minimum is obtained for channels in the ITN dataset. We note that the variance-stabilizing cofactors for the HD data set are an order of magnitude greater than those of the ITN data set. For example, the variance-stabilizing cofactor for the CD channel is 6317 in the former data set, whereas for the same channel, variance is stabilized at a cofactor of 25.1 in the latter. The primary contributing factor behind this difference is the maximum range of values in each channel. The maximum value of a fluorescence channel is 10,000 for the ITN data set, and 1,048,575 for the HD data set. Hence, variance is stabilized at higher cofactor values for the channels in the HD data set.

After identifying the optimum cofactors for each channel, we transform each sample of the ITN data set by asinh functions with the variance-stabilizing cofactors and plot the density of the transformed channels in the bottom row of Fig. 5. Similar to the HD data set (Fig. 5), the density peaks have approximately equal variance across all samples, thus confirming the homogeneity of within-population variances in one-dimensional clusters.

### Comparing flowVS with other transformation methods

We compare flowVS with three automated methods developed for transforming FC data: (a) flowTrans (b) logicle (flowCore), and (c) FCSTrans. We selected these three methods because they automatically select parameters for different transformations. As discussed earlier, flowTrans estimates the parameters of different transformations (e.g., asinh, biexponential, linlog, and Box-Cox) by maximizing the likelihood of data’s being generated from normal distributions [27]. In this paper, we chose the results of flowTrans with asinh transformation because it generated relatively better segregation of populations than the other options and is directly comparable to flowVS that also uses the asinh transformation. We generate our results by calling the flowTrans function of the Bioconductor package flowTrans. Next, we select the logicle transformation implemented in the flowCore package in Bioconductor. To estimate the parameters of logicle transformation, we use the estimateLogicle function of the flowCore package. Finally, FCSTrans also uses the logicle transformation. We obtained the R source code of FCSTrans from http://sourceforge.net/projects/immportflock/files/FCSTrans.

**Fig. 6:**
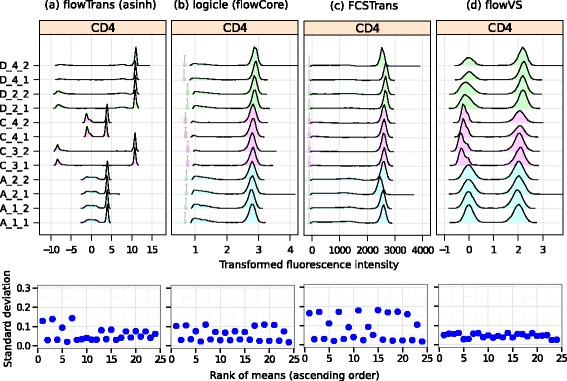
Transforming CD4 channels in HD data by four transformation algorithms. The top row shows the density plots after the data are optimally transformed by different transformations. The bottom row shows the standard deviation of density peaks against the rank of MFI

The top row of Fig. 6 shows the densities of the transformed CD4 channel of the HD data set after the samples are transformed by four methods using their optimum parameters. From visual inspection, we observe that the logicle and FCSTrans stabilize variance of the CD4^+^ and CD4^-^ populations separately. However, these two methods do not stabilize variances across CD4^+^ and CD4^-^ populations. flowTrans fails to converge for six samples (all samples from subject D and day 3 samples from subject C) and uses default cofactor=1 for these samples. Hence, the peaks transformed by flowTrans are on different scales, and they are hard to compare against each other. By contrast, flowVS stabilizes variance across all peaks of CD4 channels, including CD4^+^ and CD4^-^ populations. Furthermore, flowVS selects a single cofactor for a channel across all samples in a data set, whereas flowTrans selects different parameters for different samples. Thus, populations are more comparable after data are transformed by flowVS.

Next, we quantitatively compare the stability of variance across multiple transformation methods. This comparison, however, can not be performed on the actual transformed data because different transformations convert data to different scales. Hence, we convert each transformed channel *z* to [0,1] scale by rescaling each element *z*_*i*_ with the following equation (*z*_*i*_− min{*z*})/(max{*z*}− min{*z*}). For each transformation, we identify the density peaks in the converted CD4 channel and plot standard deviations of density peaks against their ranks of MFI in the bottom row of Fig. 6. Here we use rank of the means, instead of actual means, to distribute the points evenly along the x-axis. We observe that all four transformations are able to eliminate the systematic dependence of variance on mean, which is typically observed in untransformed fluorescence data, such as in Fig. 1. Therefore, these transformations have inherent ability to stabilize variance, mostly owing to the properties of the underlying asinh and logicle transformations. However, flowVS is able to stabilize variance more evenly than other transformations, as can be seen in the bottom right plot in Fig. 6.

**Fig. 7:**
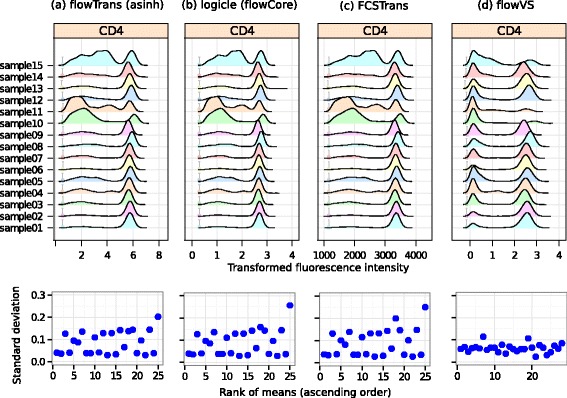
Transforming CD4 channels in ITN data by four transformation algorithms. The top row shows the density plots after the data are optimally transformed by different transformations. The bottom row shows the standard deviation of density peaks against the rank of MFI

The comparison of different transformations on the ITN data set is shown in Fig. 7. As before, the top row shows the densities of the transformed CD4 channel and the bottom row plots the standard deviations of the density peaks. As with the HD data set, flowVS stabilizes variance more evenly than other methods.

### Normality of the variance-stabilized clusters

**Fig. 8:**
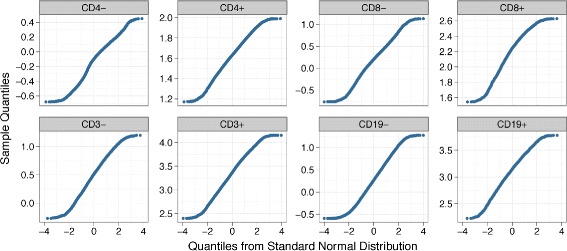
The Q-Q plots for the eight 1-D clusters obtained from a representative sample in the HD data set. Every Q-Q plot shows linearity in the central part, except for a little deviation at the end, indicating that the clusters approximately follow normal distributions with heavier tails

Bartlett’s test assumes that the cell populations are normally distributed and is sensitive to departures from normality. Density peaks (1-D cell populations) in data sets that we have studied approximately follow normal distributions. This normality assumption is typical for many FC data sets as well. Hence, a VS approach based on Bartlett’s test is expected to work well for most FC data sets. For example, in Fig. 8, we show the normality of cell populations in a representative sample of the HD data set with quantile-quantile plots (Q-Q plots) [43] of eight 1-D clusters. In each Q-Q plot, the distribution of a 1-D cluster is compared with the standard normal distribution by plotting their quantiles against each other. If a cluster is normally distributed (i.e., linearly related to the standard normal distribution), the points in the Q-Q plot lie approximately on a straight line. We observe that all eight Q-Q plots in Fig. 8 show linearity in their central parts, except for small deviations at the ends, indicating that the 1-D clusters approximately follow normal distributions with heavier tails. Therefore, flowVS based on Bartlett’s statistic works well for this data.

However, if cell populations deviate significantly from normality, we could use other likelihood ratio statistic that is less sensitive to departures from normality, such as Levene’s [44] or the Brown-Forsythe statistic [45]. In our experiments, we found Bartlett’s approach working significantly better than Levene’s, and therefore, did not show results of the latter method.

### Impact of variance stabilization in comparing cell populations

We now briefly demonstrate the impact of variance stabilization on the homogeneity of metaclusters (i.e., groups of phenotypically concordant clusters). In metacluster homogeneity evaluation, the underlying assumption is that all clusters in a metacluster represent the same cellular immunophenotype. As mentioned before, the hypothesis-testing framework may not be appropriate for the described problem, since a null hypothesis claiming that all clusters in a metacluster have equal mean is essentially always false. Moreover, when the number of cells (sampling units of the test) increases, the power of a statistical test such as a t-test or an F-test increases too. Consequently, a statistical test would inevitably detect small (i.e., statistically significant, but biologically irrelevant) differences between clusters. For example, performing a t-test with CD4^+^ cell clusters from the first and the second samples of the ITN data set, we observe *p*-values less than 10^−10^ for all transformations despite the fact that the tested cell populations are biologically identical (in an immunophenotypic sense).

Therefore, we use an effect size measure rather than *p*-values to illustrate the impact of the proposed algorithm on metacluster homogeneity. We employ the ratio of between-cluster variation (${\sigma _{b}^{2}}$) to within-cluster variation (${\sigma _{w}^{2}}$) [42]. Consider a set of *k* clusters where the *i*-th cluster containing *n*_*i*_ cells has mean *μ*_*i*_ and variance ${\sigma ^{2}_{i}}$. If *N* is the total number of cells in all clusters and *μ* is the combined mean then the ratio of ${\sigma _{b}^{2}}$ and ${\sigma _{w}^{2}}$ is computed as follows:

5 $$ \frac{{\sigma^{2}_{b}}}{{\sigma_{w}^{2}}} = \frac{\frac 1 {N-k} \sum_{i=1}^{k} (n_{i}-1)(\mu_{i} - \mu)^{2}}{\frac{1}{N-k}\sum_{i=1}^{k} {(n_{i}-1)\: {\sigma^{2}_{i}}}}.   $$

Unlike the F-test for comparing multiple clusters, the above ratio does not depend on the sample size, and it is constructed so that the increasing homogeneity of a metacluster results in a progressively smaller value of the ratio. Table 1 shows the $\frac {{\sigma ^{2}_{b}}}{{\sigma _{w}^{2}}}$ values of CD4^+^ metacluster and a biologically erroneous metacluster grouping CD4^+^ and CD4^-^ cells. The values are computed after the data are transformed by four transformations considered in this paper. In this example, the flowVS transformation gives the best homogeneity within the CD4^+^ metacluster, and an increased heterogeneity of the CD4^+^/CD4^-^ clusters mixture. Thus application of flowVS not only results in the highest homogeneity of a set of known phenotypically identical cell clusters, but also provides the best discrimination between homogeneous and heterogeneous collection of clusters. The result demonstrates that the flowVS-based variance stabilization can help in performing comparison and alignment of phenotypically identical cell populations across different samples.

**Table 1:** The ratio of between-cluster to within-cluster variations (a measure of effect size of metacluster homogeneity defined in Eq. 5) after four transformations

Cell populations	dataset	flowTrans	logicle	FCSTrans	flowVS
CD4^+^ metacluster	HD	2.8	13.70	13.42	.87
	ITN	1.3	1.55	1.54	.61
erronous CD4^-^/CD4^+^ metacluster	HD	24.40	21.63	12.64	36.77
	ITN	11.68	11.30	10.38	26.47

CD4^+^ cell populations are used in top two rows, and a mixture of CD4^+^ and CD4^-^ cell populations are used in bottom two rows. Small and large values of the ratio denote homogeneous and heterogeneous collections of clusters, respectively. In this example, flowVS transformation results in the highest homogeneity when only CD4^+^ clusters are considered, and highest heterogeneity when CD4^+^ and CD4^-^ clusters are mixed together

### Application to microarray data

The VS approach based on optimizing Bartlett’s statistic can also be used to stabilize variance in microarray data. However, the initial steps of flowVS need to be adapted for microarrays. Assume that the expression of *m* genes are measured from *N* samples in a microarray experiment. After transforming the data by the asinh function, the mean *μ*_*i*_ and variance ${\sigma ^{2}_{i}}$ of the *i*^*th*^ gene *g*_*i*_ are computed from the expressions of *g*_*i*_ in all samples. flowVS then stabilizes the variances of the genes by transforming data using the asinh function with an optimum choice of cofactor. Unlike FC, a single cofactor is selected for all genes in microarrays.

**Fig. 9:**
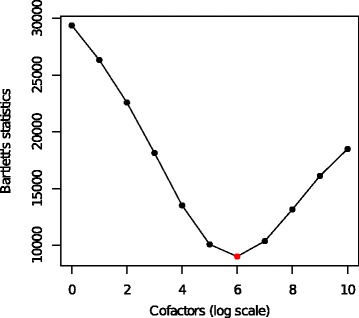
For kidney microarray data [18], flowVs selects the optimum cofactor for the asinh transformation by minimizing Bartlett’s statistic. The cofactors are shown in the natural logarithm scale

We have applied the modified flowVS to the publicly available kidney microarray data provided by Huber et al. [18]. The kidney data report the expression of 8704 genes from two neighboring parts of a kidney tumor, using cDNA microarray technology. For different values of the cofactor, flowVS transforms the kidney data with the asinh function and identifies the optimum cofactor by minimizing Bartlett’s statistic. Figure 9 shows that a minimum value of Bartlett’s statistic is obtained when the cofactor is set to exp(6) (∼400). The optimum cofactor is then used with the asinh function to transform the kidney data.

We compare the VS performance of flowVS with two software packages, VSN by Huber et al. [18] and DDHFm by Motakis et al. [46]. Similar to flowVS, VSN uses an asinh transformation whose parameters are optimized by maximizing a likelihood function [18]. DDHFm applies a data-driven Haar-Fisz transformation (HFT)[46, 47] to stabilize the variance. Both VSN and DDHFm are developed for stabilizing variance in microarray data and can not be applied to FC.

In Subfig. 10, we plot the mean and standard deviation of every gene before transforming the kidney data and after transforming it by flowVS, VSN, and DDHFm. In this figure, we have applied a loess regression to obtain smooth average curves. We observe in Subfig. 10 that the standard deviation of the untransformed kidney data increases monotonically with the mean. Both VSN and flowVs approximately stabilize the variance across all genes in this data. However, the Haar-Fisz transformation achieves good VS properties only for genes with higher levels of expression.

**Fig. 10:**
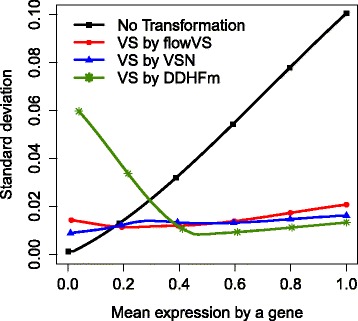
The standard deviation and mean of each gene from the kidney data are plotted before transformation and after variance stabilization by flowVs, VSN, and DDHFm. Loess regression is used to smoothen the curves

**Fig. 11:**
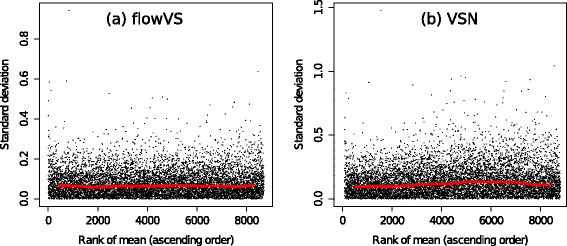
Variance stabilization of the kidney microarray data [18] by (**a**) flowVs and (**b**) VSN [18]. Each black dot plots the standard deviation of a gene against the rank of its mean. The red lines depict the running median estimator. If there is no mean-variance dependence, then the red lines should be approximately horizontal

To take a closer look at the transformed data by flowVS and VSN, we plot the variances of genes against the ranks of their means in Fig. 11. These figures are generated by the meanSdPlot function from the VSN package. Here, the ranks of means distribute the data evenly along the *x*-axis and thus make it easy to visualize the homogeneity of variances. We also show the running median estimator of standard deviation by the red lines. Both VSN and flowVS remove the mean-variance dependence because the red lines are approximately horizontal for both transformations. Hence, flowVS performs at least as well as a state-of-the-art approach developed for microarray data.

## Conclusions

We describe a variance-stabilization framework, flowVS, that removes the mean-variance correlations observed in cell populations from FC samples. This framework transforms each fluorescence channel by the asinh function whose normalization cofactor is optimally selected by Bartlett’s likelihood-ratio test. Variance homogeneity (homoskedasticity) is a desirable property for comparing populations across conditions, building metaclusters from phenotypically similar populations, and analyzing metaclusters in an ANOVA model. However, unlike the earlier approach by Hahne et al. [32], flowVS does not artificially shift populations to align them in the marker space. By stabilizing the variances, flowVS homogenizes similar cell populations and establishes the foundation of biologically meaningful metaclusters and templates.

flowVS is built on several assumptions that limit our approach. First, flowVS stabilizes variance separately in each channel. Thus it might be unable to stabilize covariances across multiple channels when they are correlated. Second, flowVS identifies 1-D density peaks and evaluates the homogeneity of populations by the likelihood-ratio test. Therefore, this algorithm might not perform well when density peaks are not easily identifiable. Third, flowVS stabilizes variance more accurately when a number of samples are simultaneously passed to the algorithm. Hence, this approach is not suitable for normalizing a single sample or stabilizing variances of sequentially arriving samples. Finally, Bartlett’s test used in flowVS assumes that the deviation from normality is relatively modest. If data deviate significantly from normality, other likelihood ratio tests can be employed, such as Levene’s test [44] or the Brown-Forsythe test [45].

flowVS operates as an independent module in the FC data analysis pipeline. It does not depend on the preprocessing algorithms applied before VS nor on the post-analysis methods such as matching, metaclustering, and classification. Hence, flowVs is capable of working with most automated clustering and metaclustering algorithms developed for flow cytometry.

## Abbreviations

asinh, inverse hyperbolic sine; FC, flow cytometry; ITN, immune tolerance network; MFI, mean fluorescence intensity; VS, variance stabilization.
